# Transcriptomic adaptation during skeletal muscle habituation to eccentric or concentric exercise training

**DOI:** 10.1038/s41598-021-03393-7

**Published:** 2021-12-14

**Authors:** Craig R. G. Willis, Colleen S. Deane, Ryan M. Ames, Joseph J. Bass, Daniel J. Wilkinson, Kenneth Smith, Bethan E. Phillips, Nathaniel J. Szewczyk, Philip J. Atherton, Timothy Etheridge

**Affiliations:** 1 Department of Sport and Health Sciences, College of Life and Environmental Sciences, University of Exeter, Exeter, EX1 2LU UK; 2 Department of Biomedical Sciences, Heritage College of Osteopathic Medicine, Ohio University, Athens, OH 45701 USA; 3 Ohio Musculoskeletal and Neurological Institute, Ohio University, Athens, OH 45701 USA; 4 Living Systems Institute, University of Exeter, Stocker Road, Exeter, EX4 4QD UK; 5 Department of Biosciences, University of Exeter, Stocker Road, Exeter, EX4 4QD UK; 6 MRC-Versus Arthritis Centre for Musculoskeletal Ageing Research and National Institute of Health Research, Nottingham Biomedical Research Centre, Royal Derby Hospital Centre, School of Medicine, University of Nottingham, Derby, DE22 3DT UK

**Keywords:** Computational biology and bioinformatics, Transcriptomics, Skeletal muscle

## Abstract

Eccentric (ECC) and concentric (CON) contractions induce distinct muscle remodelling patterns that manifest early during exercise training, the causes of which remain unclear. We examined molecular signatures of early contraction mode-specific muscle adaptation via transcriptome-wide network and secretome analyses during 2 weeks of ECC- *versus* CON-specific (downhill *versus* uphill running) exercise training (exercise ‘habituation’). Despite habituation attenuating total numbers of exercise-induced genes, functional gene-level profiles of untrained ECC or CON were largely unaltered post-habituation. Network analysis revealed 11 ECC-specific modules, including upregulated extracellular matrix and immune profiles plus downregulated mitochondrial pathways following untrained ECC. Of 3 CON-unique modules, 2 were ribosome-related and downregulated post-habituation. Across training, 376 ECC-specific and 110 CON-specific hub genes were identified, plus 45 predicted transcription factors. Secreted factors were enriched in 3 ECC- and/or CON-responsive modules, with all 3 also being under the predicted transcriptional control of SP1 and KLF4. Of 34 candidate myokine hubs, 1 was also predicted to have elevated expression in skeletal muscle *versus* other tissues: *THBS4*, of a secretome-enriched module upregulated after untrained ECC. In conclusion, distinct untrained ECC and CON transcriptional responses are dampened after habituation without substantially shifting molecular functional profiles, providing new mechanistic candidates into contraction-mode specific muscle regulation.

## Introduction

Starting a new exercise regime initiates a highly dynamic period for skeletal muscle. The first unaccustomed bout of strenuous activity can cause muscle ultrastructural damage and declines in functional capacity^1^. Muscle then rapidly recovers within ~ 7 days and such remodelling, in turn, confers resilience against subsequent exercise-induced damage (the ‘repeated bout effect’)^2^. Over ~ 2–3 weeks, this initial period of intense remodelling translates to rapid gains in muscle composition and performance, doing so in a manner specific to the mechanical and metabolic demands of the exercise programme^3^. Thereafter, post-exercise muscle turnover abates and muscle structural and functional improvements progress comparatively slowly^4^. This biphasic response likely reflects rapid early repair of damaged proteins and gross muscle remodelling events, *versus* longer-term adaptive processes that become increasingly exercise-mode specific^5^. Thus, the initial weeks of training represent a key window for optimising exercise adaptation.

Illustrating the early plasticity of muscle are specialised adaptations to lengthening ‘eccentric’ (ECC) or shortening ‘concentric’ (CON) contractions. Appearing early (≤ 4 weeks) into training, ECC associates with a greater initial damage-repair profile and chronic increases in muscle fascicle length, whereas CON induces minimal acute damage and chronically favours increased fiber pennation angle^6–8^. Whilst presumably reflecting the dominant ECC-induced mechanical load compared to a higher CON-related metabolic stress, the mechanisms responsible for these distinct adaptive responses are unclear. Transcriptionally, a single unaccustomed ECC resistance exercise bout appears unique from CON in only a minor, heat shock protein-related manner^9^, in line with specific post-ECC demands to stabilise and repair damaged proteins. Targeted analyses also indicate a greater acute ECC-induced muscle anabolic^10^, inflammatory^11^ and satellite cell^12^ signalling response than CON. Nonetheless, global analyses indicate that acute ECC and CON induce largely overlapping transcriptomic signatures in exercise-naïve muscle^9^, likely reflective of a generalised global repair/remodelling molecular response to unaccustomed exercise. Discordant molecular signatures might therefore emerge over the early stages of repeated exercise bouts as muscle develops ECC/CON phenotypic specificity^6^. Nevertheless, while acute transcriptional changes during training have been well classified for more traditional exercise modalities (i.e., conventional resistance or endurance exercise models; e.g.,^13–18^), they remain to be fully characterised in the context of ECC *versus* CON.

To identify novel molecular drivers of muscles’ early habituation to exercise training, transcriptomics-driven network analysis represents a powerful tool that accounts for the inherent complexity of biological systems, beyond reductionist or traditional differential gene expression analyses alone^19–21^. Global mRNA screens can also be used to identify putative myokines^22,23^: muscle-derived secretory factors thought to systemically govern multi-tissue crosstalk and facilitate whole-body exercise adaptation^24–26^. The muscle ‘secretome’ comprises thousands of myokines^27^ likely predominantly serving autocrine/paracrine actions that assist muscle remodelling through diverse anabolic, inflammatory, mitochondrial, extracellular matrix (ECM) and angiogenic mechanisms^24,28^. This functional diversity appears refined to meet the precise mechanical/metabolic demands of exercise^24,27,28^, thus the muscle secretome might feasibly perform important roles in contraction mode-specific remodelling. Nonetheless, current understanding of the contraction mode-regulated secretome is limited to a small number of well-defined myokines (e.g., IL-6) and mostly in blood^11,29–33^. Since secreted proteins represent a significant class of efficacious pharmaceutical targets^34^, better knowledge of how the intrinsically dense muscle secretome is regulated by contraction mode has important clinical implications, for example the development of exercise mimetics^26,27^.

We therefore determined how acute ECC or CON muscle transcriptomic profiles are modified by habituation to ECC- or CON-specific training. Using the power of network analysis, we also identify putative muscle intrinsic and secretory regulators that might associate with unique muscle phenotypic adaptations to specific contractile demands.

## Methods

### Subject characteristics

Sixteen healthy young adult males (mean ± SD: age, 23 ± 5 y; body mass index, 24 ± 3 kg m^−2^) volunteered to partake in this study. All participants were exercise-naïve, defined as not performing planned weekly physical exercise (excluding activities of daily living, such as walking) and having no history of regular, structured exercise training within the previous ~ 12 months. Participants were also free from consuming tobacco-containing products, dietary supplements, or any other form of medication (including anti-inflammatory drugs) and were asked to remain so during the study. Experimental procedures were approved by the Sport and Health Sciences Ethics Committee at the University of Exeter and performed in accordance with the relevant guidelines and regulations. Written informed consent was obtained from all volunteers prior to their participation.

### Experimental procedures

**Figure 1:**
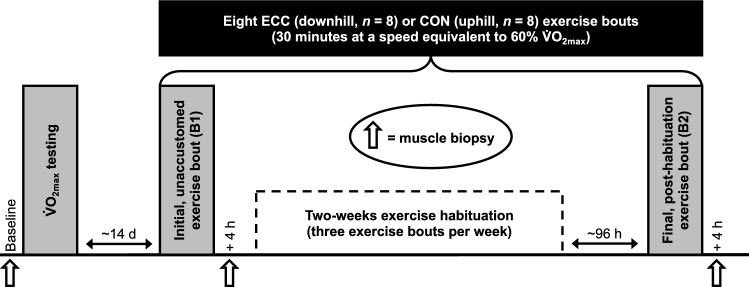
Schematic diagram of the study design. Participants completed a total of either eight ECC (downhill running, *n* = 8) or CON (uphill running, *n* = 8) exercise bouts, of which consisted of: (i) an initial, unaccustomed bout (B1); (ii) 2-weeks of exercise habituation (three bouts per week), and; (iii) a final, post-habituation bout (B2). Muscle biopsies were collected at baseline, 4 h following B1 and 4 h following B2.

Participants visited the laboratory on nine main occasions (Fig. 1). On *visit 1*, a baseline muscle sample was collected from the *m. vastus lateralis* of the non-dominant leg under local anaesthesia (2% Lidocaine) using the percutaneous Bergström needle technique with suction^35^. Muscle tissue was blotted on gauze to remove excess blood, snap frozen in liquid nitrogen and stored at − 80 °C until further analysis. Participants then completed an incremental treadmill test for the determination of maximal oxygen uptake (V̇O_2max_) as described^36^. Briefly, participants completed 6 min of light jogging at 7 km h^−1^, after which the speed was increased by 1 km h^−1^ each minute until volitional exhaustion, with the treadmill gradient maintained at + 1%. Pulmonary gas exchange measurements were recorded continuously using a metabolic stress test system (Cortex Metalyzer 2B, Cortex Biophysik, Germany) and averaged over 10 s intervals. V̇O_2max_ was subsequently taken as the highest average oxygen uptake for a given 10 s period during the incremental treadmill test.

For *visit 2*, participants were randomly assigned to undertake an initial, unaccustomed bout (B1) of ECC- or CON-specific exercise in the form of downhill (*n* = 8) or uphill (*n* = 8) treadmill running, respectively, which took place ~ 2 weeks following *visit 1* to limit any residual molecular impact of V̇O_2max_ testing. Each ECC or CON bout comprised 30 min of treadmill running (3 × 10-min bouts with 2-min active rest (walking) intervals) at a speed equivalent to 60% V̇O_2max_, with the treadmill gradient set at -10% for ECC and + 10% for CON. A further muscle biopsy was collected 4 h post-exercise as described above. Across *visits 3–8*, participants completed the same 30-min ECC or CON protocol as above on six more occasions over a 2-week training habituation period (3× sessions per week), with each session separated by at least one rest day. For *visit 9*, approximately 96 h following the last exercise training session, participants performed a final, post-habituation bout (B2) of the same ECC or CON exercise, after which another 4 h post-exercise muscle biopsy was again collected as described. Thus, exactly three biopsies were collected per participant, and a total of 48 biopsies obtained overall (eight biopsies per timepoint per group). Muscle samples were obtained from separate incision sites spaced ~ 3 cm apart in order to minimise any effect of the previous biopsy collection(s)^37^. Main experimental visits (*visit 1*, *visit 2* and *visit 9*) were carried out in the morning at a consistent time (~ 8:00 h), with participants in the post-absorptive state (≥ 10 h overnight fasting period) and having refrained from caffeine or alcohol consumption for 24 h prior to their arrival. All exercise bouts were performed on the same slat-belt treadmill (Woodway, Weil am Rhein, Germany).

### Generation and pre-processing of RNA-sequencing data

All RNA extraction, library preparation and next generation sequencing was performed by the Beijing Genomics Institute, Hong Kong. In brief, the RNA concentration and integrity of each sample was assessed using the Agilent 2100 Bioanalyzer system (Agilent Technologies). All forty-eight samples met the requirements for library preparation (RNA integrity number ≥ 7.0, 28S/18S ≥ 1.0) and were sequenced to generate 50 base pair single-end reads using the BGISEQ-500 platform. The quality of cleaned reads was consequently established using FastQC (Babraham Bioinformatics). Since all reads were deemed to be of good quality (no over-represented sequences or adapter sequences and median per base quality scores always > 30), no additional filtering or trimming of reads was performed. Reads were subsequently aligned to the human reference genome (GRCh38.p13 Primary Assembly, Ensembl) using HISAT2^38^, with reads mapping to known exons then counted in an un-stranded manner using featureCounts^39^ and with the human genome annotation used as a reference (GRCh38.p13, Ensembl). Lowly expressed genes across samples (read count < 10 in at least 50% of all samples) were consequently removed, leaving a total of 17,411 genes across the forty-eight samples for use in downstream analyses.

### Traditional gene-level expression analysis

Differential gene-level expression analysis was undertaken using the DESeq2 package for R^40^ and in accordance with developers recommendations for mixed-design experiments (DESeq2 vignette—‘Group-specific condition effects, individuals nested within groups’). In brief, size factors and dispersion estimates were first calculated and used to fit a generalised linear model to each gene, with modelled read counts following a negative binomial distribution and a model matrix accounting for both between and within individual effects as per the developers guidance. Wald tests were then used to identify significant differentially expressed genes following B1 or B2 (relative to baseline) for each group. The Benjamini-Hochberg (BH) procedure was used to control for false discovery rate (FDR). Differentially expressed genes in each case were defined as those with a BH-corrected *P* < 0.05. The rank-rank hypergeometric overlap (RRHO) algorithm^41^ was then used to determine common and uniquely regulated genes both across exercise bouts within each group as well as between groups following each exercise bout, with genes ranked on sign of differential expression multiplied by the negative log10 of their BH-corrected *P*-value. For each comparison, commonly regulated genes were defined as those significantly differentially expressed in both given conditions *and* present within the optimal overlapping gene set between the given conditions. Uniquely regulated genes in each case were then defined as those significantly differentially expressed in a single given condition and *not* present within the optimal overlapping gene set between the given conditions.

### Gene co-expression network construction and module detection

Gene-wise network construction was undertaken using the weighted gene co-expression network analysis (WGCNA) package implemented in R^42^. Prior to network construction, raw read counts of filtered genes were normalised using a variance-stabilising transformation^43^ in order to attain homoscedastic expression data. A signed weighted adjacency matrix quantifying the connection strength between each pair of genes was first derived as *Adj* =|0.5 × (1 + *Corr*)|^*ß*^, where *Corr* is the matrix of Pearson’s correlation coefficients that indicate the degree of similarity in expression pattern between any two given genes in the dataset. The exponent *ß* was chosen in accordance with the scale-free topology criterion^44^ as the lowest integer for which the corresponding scale-free topology fitting index metric achieved an appropriately high value (≥ 0.9). A relative interconnectedness measure for each gene pair (dissimilarity topological overlap) was then quantified from the adjacency matrix and subject to average linkage hierarchical clustering to generate a network tree. Network modules were consequently determined from the network tree via the *cutreeDynamic* algorithm^45^, using a minimum module size threshold of 30. Modules were subsequently merged if their composite expression (module eigengene; 1st principal component of module gene expression) was very similar (eigengene correlation coefficient ≥ 0.75). Modules were assigned a unique numerical label ‘M*i*’ for identification, with all genes able to be trivially clustered.

### Establishing module differential regulation

The effects of time and contraction mode on module expression patterns were established by undertaking differential analysis of module eigengenes using the limma package for R^46^, in line with developers recommendations for mixed design experiments (limma user guide—‘9.7 Multi-level Experiments’). Specifically, a linear mixed-effects model was first fitted to each modules eigengene, with a group means parameterisation of experimental condition (i.e., all possible mode-time permutations) included as a fixed effect, and participant included as a random effect to account for the correlation between samples from the same individual. Empirical Bayes-moderated paired *t*-tests were then applied to determine module differential regulation following B1 or B2 (relative to baseline) for each group. Statistical significance was accepted in instances where a BH-corrected *P* < 0.05 was observed.

### Functional annotation of exercise gene changes

The functional characteristics of differentially expressed genes and differentially regulated network modules were derived by testing their comprising gene lists for enrichment of Gene Ontology (GO) terms. Analyses were undertaken using the clusterProfiler package in R^47^, with the corresponding background gene list used in each instance being the genes used as input during differential expression testing/ network construction. Each of the three separate GO categories (biological process, cellular component, molecular function) were considered during analyses and enriched terms selected as those with a BH-corrected *P*-value < 0.05 enriched for at least 2 genes.

### Network-driven prediction of key mechanistic targets

Candidate regulatory molecules were determined via two approaches. First, key molecular drivers within differentially regulated network modules (module ‘hub’ genes) were established as based on their scaled within-module connectivity (kIM)^48^, such that genes within a given module with a scaled kIM value ≥ 0.7 were classified as modular hubs^49^. Second, putative transcriptional regulators of differentially regulated modules were predicted by testing their corresponding gene lists for enriched transcription factor (TF) binding site(s) (TFBS) in the 5 kilobase upstream/downstream region encompassing transcription start sites. Within such analyses, the oPOSSUM-3 webserver^50^ was used to query JASPAR core vertebrae profiles with a minimum specificity of 8 bits meeting a conservation cut-off of 0.4 and similarity matrix score threshold of 85%, with the corresponding background gene list in each case constituting the genes inputted during network construction. Of note, large modules (> 2000 genes) were represented by their top 75% most connected genes. Enriched TFBS were selected as those with a corresponding Z-score and Fisher score ≥ the mean + 1.5 SD of their respective distributions^49^.

### Cross-network comparison of contraction-induced expression patterns

In previous work, we applied WGCNA to RNA-sequencing data that was generated from, in part, young (18–30 y) human skeletal muscle before and following a single unaccustomed bout of either isolated CON or isolated ECC contractions^49^. Since both our previous and current work recruited similar cohorts of younger volunteers (i.e., healthy, exercise-naïve males), we chose to compare module regulation patterns across both associated networks, on the premise that modules across the two networks with similar gene compositions *and* differential regulation patterns might represent molecular networks reproducibly regulated by contraction mode and hence particularly strong mechanistic candidates of contraction-induced muscle remodelling. We first mapped the modules of our previously constructed network to those of our newly constructed network, labelled herein as the ‘single-bout’ and ‘multi-bout’ study networks, respectively. Specifically, module pairs across networks with similar gene compositions were identified using the WGCNA *matchLabels* function (based on genes intersecting across networks), the underlying procedures of which are described in detail elsewhere^51^. We then determined single-bout network modules regulated by ECC and/or CON in younger muscle that best map to a contraction-regulated module in the multi-bout network. Such module pairs were further probed for the presence of overlapping module hub genes.

### Network-driven identification of muscle secretome networks and candidate myokines

To explore contraction mode secretome regulation in muscle, we utilised the human secretome as characterised by the Human Protein Atlas (version 20.1); a list of 1708 genes conservatively predicted to have at least one secreted protein variant using a compendium of different bioinformatic tools^34^. Briefly, this list comprises of genes having at least one protein variant: (i) predicted to contain a signal peptide based on a majority decision method of three signal peptide prediction tools (SignalP 4.0, Phobius, SPOCTOPUS) with no predicted transmembrane region based on majority decision method of seven transmembrane region prediction tools (MEMSAT3, MEMSAT-SVM, Phobius, SCAMPI, SPOCTOPUS, THUMBUP, TMHMM), or otherwise annotated under the UniProt keyword ‘Secreted’, and; (ii) not annotated as intracellular and/or membrane-bound (e.g., endoplasmic reticulum or Golgi residing, mitochondrial, lysosomal, membrane-associated, etc.). We then identified putative exercise-induced muscle secretome-related networks by establishing differentially regulated modules enriched for secretome components. In which case, enrichment was assessed using Fisher’s exact test, with statistical significance accepted when *P* < 0.05. Key myokine candidates of contraction mode and/or short-term training responses were further deduced by screening hub genes of each differentially regulated module for secretome components. As a complementary measure, we also considered which of these myokine candidates might show elevated expression in skeletal muscle *versus* other tissues^23^ using the Human Protein Atlas (version 20.1) skeletal muscle-specific proteome database^34^.

## Results

### Effect of exercise habituation on ECC or CON individual gene responses

**Figure 2:**
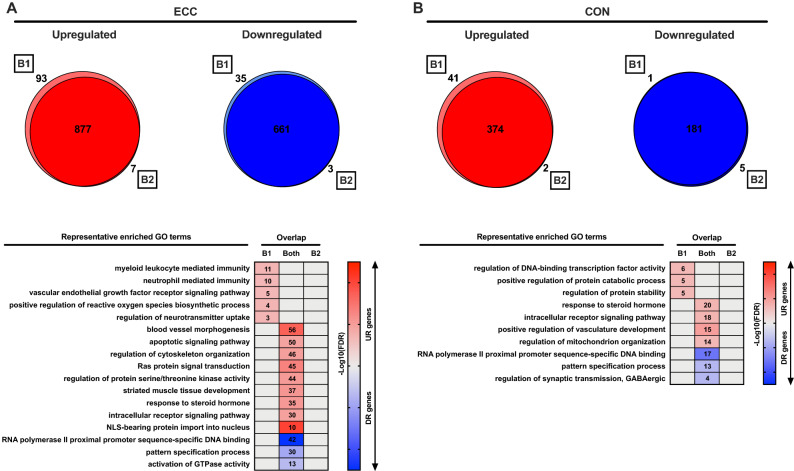
Training-(in)dependent gene-level responses to acute ECC and CON. (**A**) Venn diagrams depicting the degree of overlap between genes upregulated (UR) or downregulated (DR) by acute ECC in untrained *versus* exercise habituated muscle, along with a heatmap illustrating representative enriched GO terms for each corresponding unique/common gene set. Number of genes enriched for a given term are provided within associated boxes of the heatmap. Strength of colour shading depicts magnitude of enrichment significance, given by the negative log10 of that term’s enrichment FDR *P*-value (with darker shading analogous with a stronger FDR *P*-value). (**B**) As with (**A**), but for genes regulated by acute CON in untrained *versus* exercise habituated muscle. B1 = initial, unaccustomed exercise bout. B2 = final, post-habituation exercise bout.

We first considered how individual gene expression changes are altered after a short-term, 2-week period of either ECC- or CON-specific exercise training habituation. Overall, ECC exercise altered the expression of at least ~ twice as many genes as CON, in both the untrained and habituated state (Table 1). Habituated muscle also displayed a drastically attenuated total number of differentially expressed genes *versus* untrained muscle, for both ECC and CON (Table 1). However, when integrating the RRHO algorithm, the transcriptional profiles to both ECC and CON were largely unaltered by 2-weeks habituation (Fig. 2A,B). Nonetheless, whilst virtually no genes were uniquely regulated after habituation to ECC or CON, minor transcriptional profiles specific to untrained exercise responses were identified (Fig. 2A,B). These untrained-specific profiles mostly associated with upregulated genes, enriched post-ECC for immune system-related processes (Fig. 2A), and post-CON for protein stability and catabolism pathways (Fig. 2B). Of the genes that were altered regardless of training status, both ECC and CON associated with a pattern of upregulated steroid hormone and angiogenesis processes, and downregulated RNA polymerase II transcription (Fig. 2A,B). However, the generic ECC response was further characterised by upregulation of genes involved in cytoskeletal organisation and muscle tissue development (Fig. 2A), whilst the generic CON response included upregulation of mitochondrial organisation genes (Fig. 2B). No enriched terms were found for the small number of genes uniquely regulated post-exercise in habituated muscle in either case.

**Table 1:** Total numbers of significant differentially expressed genes 4 h following acute exercise in untrained (B1) and habituated (B2) muscle.

	ECC		CON	
	B1	B2	B1	B2
Upregulated	2188	1043	908	533
Downregulated	1933	779	396	443

### Effect of contraction mode on untrained and habituated individual gene expression

**Figure 3:**
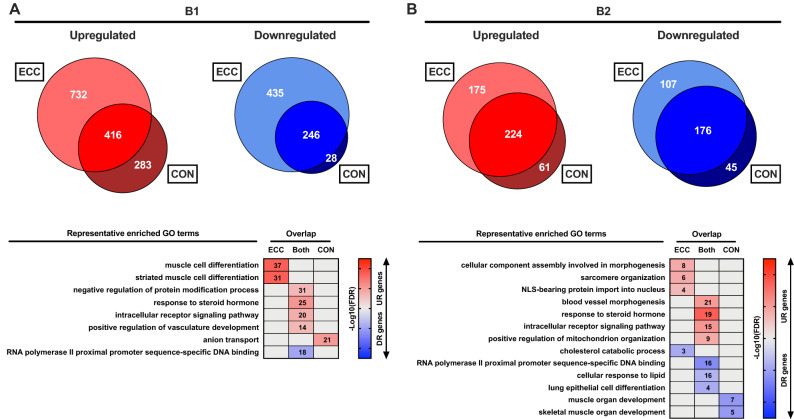
Contraction mode-(in)dependent gene-level responses in untrained and habituated muscle. (**A**) Venn diagrams depicting the degree of overlap between genes upregulated (UR) or downregulated (DR) by acute ECC *versus* CON in untrained muscle, along with a heatmap illustrating representative enriched GO terms for each corresponding unique/common gene set. Number of genes enriched for a given term are provided within associated boxes of the heatmap. Strength of colour shading depicts magnitude of enrichment significance, given by the negative log10 of that term’s enrichment FDR *P*-value (with darker shading analogous with a stronger FDR *P*-value). (**B**) As with (**A**), but for genes regulated by acute ECC *versus* CON in exercise habituated muscle. B1 = initial, unaccustomed exercise bout. B2 = final, post-habituation exercise bout.

We observed clear contraction mode-dependent responses to exercise in both untrained and exercise habituated muscle (Fig. 3A,B). In untrained muscle, upregulated genes involved in striated muscle development were unique to ECC, and upregulated genes associated with anion transport were CON-specific (Fig. 3A). However, there was a lack of ontological enrichment for uniquely downregulated genes for either ECC or CON in the untrained state. In habituated muscle, genes uniquely upregulated post-ECC remained structurally related, including those involved in sarcomere organisation and cellular component morphogenesis processes, and genes uniquely downregulated post-ECC were enriched for cholesterol catabolism pathways (Fig. 3B). Whilst no enriched terms were identified for genes uniquely upregulated post-CON after habituation, uniquely downregulated genes were enriched for muscle organ development processes (Fig. 3B). Despite this contraction mode transcriptional specificity, a large number of genes were commonly regulated by ECC and CON in untrained and/or habituated muscle, with both untrained and habituated muscle upregulated genes enriched for angiogenesis and steroid hormone signalling terms (Fig. 3A,B). Moreover, of the 252 gene symbols commonly regulated by ECC and CON in untrained muscle in our previous ‘single-bout’ study^9^, 234 (93%) were identified in the current work and 97 of these (41%) were also found to be commonly regulated by ECC and CON in untrained muscle herein. Full lists of common/unique genes from the current work and their enriched GO terms are given in Supplementary File 1.

### Network analysis of ECC and CON transcriptomes in untrained and habituated muscle

**Figure 4:**
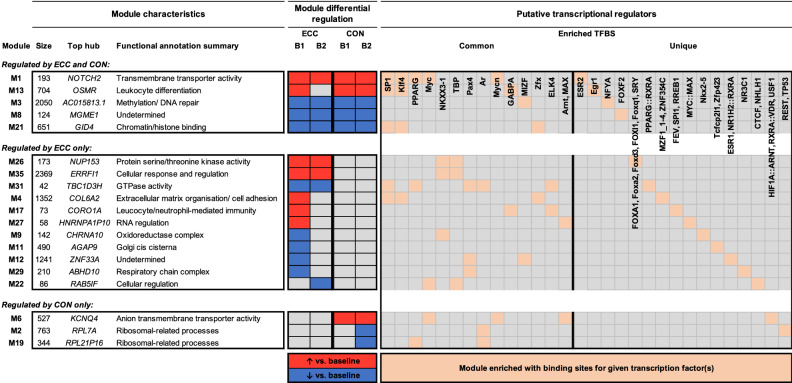
Network analysis of transcriptomic responses to ECC and CON in untrained and habituated muscle. Modules shown are those with a co-expression profile responsive to acute ECC and/or CON in untrained muscle, exercise habituated muscle, or both. Red and blue shading denote significant post-exercise upregulation and downregulation relative to baseline, respectively (FDR < 5%). Also provided is each given module’s top ranked hub gene, its functional annotation summary as based on enriched GO terms, and its enriched TFBS. B1 = initial, unaccustomed exercise bout. B2 = final, post-habituation exercise bout.

We next performed data-driven network analysis by modelling interactions among genes to construct a co-expression network, using WGCNA^44^. A total of thirty-seven distinct groups of co-regulated genes (network ‘modules’) were identified, nineteen of which displayed a contraction responsive expression profile in untrained and/or exercise-habituated muscle (BH-corrected *P* < 0.05; Fig. 4, Supplementary File 2). The majority of these nineteen modules (*n* = 11) were regulated in an ECC-dependent manner. In addition to biological themes congruent with our gene-level analysis (e.g., immune-related (M17) and ECM-related (M4) gene signatures specifically post-ECC in untrained muscle), network analysis identified several additional molecular features of the untrained ECC response, including the downregulation of mitochondria-related modules (M9, M29). Only three modules were regulated in a CON-specific manner. Two were downregulated post-CON in habituated muscle and enriched for ribosome-related genes (M2, M19), and one anion transporter activity-related module was upregulated by CON in both untrained and habituated muscle (M6). Several modules also represented generalised exercise responsive molecular networks (i.e., regulated by ECC and CON in untrained and habituated muscle). These included an upregulated module enriched for transmembrane transporter activity genes (M1), and downregulated modules enriched for DNA methylation- and chromatin/histone binding-related genes (modules M3 and M21, respectively).

A key advantage of network analysis is the systematic deduction of candidate regulatory molecules of muscle adaptation^49^. As such, we applied two biologically-relevant data reduction techniques to each differentially regulated module, to detect promising molecular candidates of contraction mode- and/or training status-dependent muscle adaptations, namely: (i) hub gene identification^48^, and; (ii) predictive TF analysis^50^. In which case, we identified a base list of candidate regulatory molecules (656 hub genes and 45 putative transcriptional regulators of co-expression) putatively involved in the early stages of muscle habituation to ECC and/or CON exercise training (top ranked hub gene and all enriched TFBS of each pertinent module given in Fig. 4, full hub lists available in Supplementary File 2). For example, the SP1 TF involved in myoblast differentiation^52^ was returned for predictive control of several ECC and CON modules (Fig. 4). Similarly, KLF4, part of the Kruppel-like factor family of TFs which contribute to lipid metabolic homeostasis and myotube maturation^53^, also predictively regulated several ECC and CON responsive network modules (Fig. 4). Interestingly, ECC per se also uniquely downregulated a module in which hub genes were exclusively TBC1D3 family members (M31; see Supplementary File 2), which act as GTPase activating proteins for RAB5^54^.

**Figure 5:**
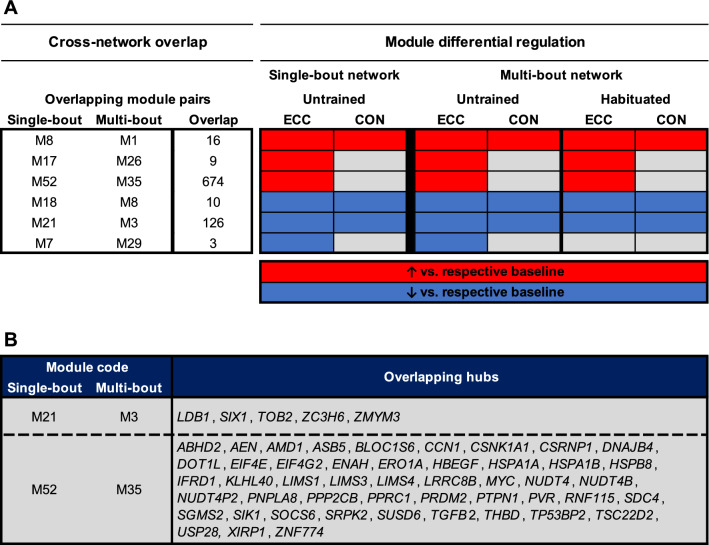
Cross-network comparison of transcriptomic responses to ECC and CON. (**A**) Modules differentially regulated by ECC and/or CON in untrained younger muscle from our previous work (‘single-bout’ network; see Willis et al.^49^) which map to a module differentially regulated by ECC and/or CON in our current work (‘multi-bout’ network). Red and blue shading denote significant post-exercise upregulation and downregulation relative to corresponding baselines, respectively (FDR < 5% in all cases). (**B**) Overlapping hub genes (if any) for each module pair defined in (**A**).

To determine modules and hub genes reproducibly regulated by ECC or CON exercise, we further cross-referenced our current network (the ‘multi-bout’ network) with our previous network of untrained ECC and CON responses (the ‘single-bout’ network)^49^. In the previous single-bout network, ten modules were regulated by ECC and/or CON exercise in young, untrained muscle (see Figure 3 of Willis et al.^49^). Of these, seven were recapitulated in the present multi-bout network (i.e., had a significant number of overlapping genes with one of the multi-bout network modules), from which six were consequently found to best map to one of the current differentially regulated modules (Fig. 5A). Specifically, three overlapping module pairs elucidated regulation by exercise per se (i.e., independent of contraction mode and habituation), two module pairs demonstrated upregulation by ECC alone, and one module pair showed downregulation by ECC in the untrained state (Fig. 5A). Additionally, two module pairs had multiple overlapping hub genes (Fig. 5B). Firstly, the M21(single-bout)/M3(multi-bout) module pair showed downregulation across ECC and CON and shared five common hub genes, including several LIM-domain and zinc-finger containing genes (e.g., *LDB1*, *ZC3H6*, *ZMYM3*) (Fig. 5B). Secondly, the M52(single-bout)/M35(multi-bout) module pair was upregulated by ECC alone and shared forty-eight common hub genes, including several established regulators of skeletal muscle (e.g., *EIF4E*, *MYC*, *TGFB2*) (Fig. 5B).

### Network-driven prediction of ECC/CON- and habituation-responsive myokines

**Figure 6:**
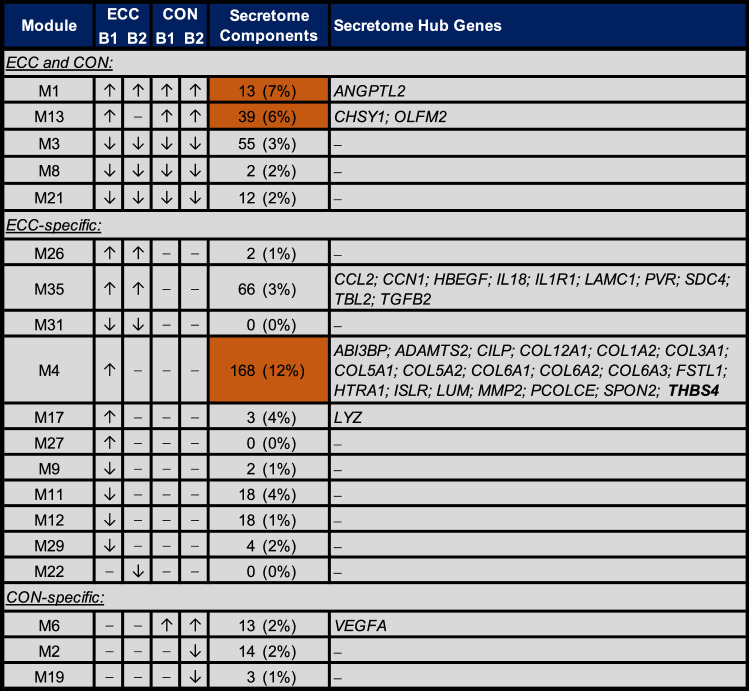
Network-driven secretome analysis of ECC- and CON-exercised muscle. Shown are those modules differentially regulated by ECC and/or CON in untrained muscle, exercise habituated muscle, or both (as outlined in Fig. 4). Given in each case are both the total number and proportion (%) of module genes that are predicted to encode a putative secretome component, and module hub genes (if any) that represent candidate myokine targets. Orange shading denotes a significant enrichment of module genes for putative secretome components, with bold font indicating those myokine candidates predicted to have elevated expression in skeletal muscle tissue *versus* other tissues. B1 = initial, unaccustomed exercise bout. B2 = final, post-habituation exercise bout.

We further capitalised on the predictive capacity of network analysis to identify prominent muscle secretome networks and key myokine targets of muscle responses to contraction mode and habituation. Of the nineteen contraction responsive modules identified herein, three were significantly enriched for putative human secretome components (Fisher’s exact *P* < 0.05) and may thus potentially represent prominent myokine-related muscle regulatory pathways (Fig. 6, Supplementary File 3). One of these modules was specifically upregulated by ECC in untrained muscle and enriched for genes involved in ECM organisation/ cell adhesion (M4). The remaining two were a transmembrane transporter activity-related module upregulated by ECC and CON in both untrained and habituated muscle (M1), and a leukocyte differentiation-related module upregulated by untrained and habituated CON and untrained ECC (M13). Cross-comparison of putative transcriptional regulators of these secretome-enriched modules identified all three to be under the predicted control of the SP1 and KLF4 TFs (Fig. 4).

We further screened the complete hub gene lists of contraction responsive modules for putative human secretome components, to derive key myokine candidates. Of the total 656 hub genes, 34 identified as potential myokines (Fig. 6, Supplementary File 3). Notably, the vast majority (*n* = 30) of these were hub genes of modules specifically regulated by ECC exercise. These, in turn, were almost exclusively contained within two ECC-specific modules: (i) the ECM/ cell adhesion module (M4) upregulated by untrained ECC that also displayed enrichment for secretome components, and; (ii) a general cellular response/regulation module (M35) upregulated by ECC in both untrained and habituated muscle (Fig. 6). Just a single CON-specific hub gene (*VEGFA*) was identified among putative secretome components, contained within the anion transporter-related module upregulated post-CON in both untrained and habituated muscle (M6). Lastly, we screened the 34 hub myokine candidates for those that show elevated expression in skeletal muscle *versus* other tissue types. In which case, just 1 of the 34 primary myokine candidates was predicted to be skeletal muscle ‘elevated’, namely: *THBS4*, a thrombospondin glycoprotein involved in cell–cell interactions and maintaining muscle integrity^55,56^, contained within the secretome-enriched module specifically upregulated after untrained ECC (M4) (Fig. 6).

## Discussion

ECC and CON contractions induce distinct muscle remodelling patterns that emerge early (≤ 4 weeks) into a new training programme^6,57^. Incompletely understood, however, are the initial mechanisms governing such contraction mode-dependent muscle regulation. The present study thus investigated how contraction mode and habituation to training interact to influence muscle transcriptomic responses to exercise. We report that the untrained response to both ECC and CON associates with larger and functionally distinct transcriptional responses compared to post-habituation. In turn, habituated muscle transcriptomic profiles minimally diverge from untrained for both ECC and CON. Contraction mode specificity occurred in both the untrained and habituated state, and network analysis identified several new molecular candidates of ECC *versus* CON muscle responses to short-term training.

Unaccustomed exercise requires muscle to rapidly remodel from widespread ultrastructural disruption^1^, which abates as muscle becomes more resilient to exercise-induced damage (i.e., the ‘repeated bout effect’)^2^. Our observation of drastic attenuation of the post-exercise transcriptome implies that this diminishing structural/functional remodelling over repeated exercise bouts is reflected, and perhaps orchestrated, at the transcriptional level. Congruent with this, recent analysis of ~ 50 selectively chosen ‘exercise responsive’ genes in muscle found heightened expression during the first week of ECC + CON or isolated CON training, which diminished beyond 4 weeks of training^58^. Similarly, microarray/RNA-seq studies across other exercise modalities (including conventional resistance or endurance exercise models) also commonly report an increased transcriptional sensitivity of exercised muscle in the untrained *versus* trained state^13,16,18,59^. It is therefore unsurprising that, within contraction mode, we found very few unique post-habituation gene signatures. Whilst the mechanisms underpinning this phenomenon remain undefined, widespread epigenetic changes specifically in myonuclei might promote the more ‘refined’ transcriptional response of acutely exercised muscle after training^60^ that we and others have observed. Regardless, rapidly subsiding transcriptome-wide gene induction during the dynamic early weeks of exercise training thus presents as a robust general feature of remodelling muscle.

Unaccustomed ECC induces greater structural and functional muscle damage than unaccustomed CON^8,12^. Moreover, chronic ECC *versus* CON associate with divergent muscle phenotypic adaptations that emerge early (≤ 4 weeks) into training, despite comparable muscle mass and strength gains^6,7,57^. Accordingly, we find distinct functional transcriptional profiles characterising ECC and CON, which are more pronounced in untrained muscle but persist after habituation. Similar to previous smaller scale analysis^58^, ECC provoked a heightened transcriptional response than CON in both the untrained and early habituated state, which manifested as specific molecular functional signatures. Untrained ECC-specific gene signatures centred on upregulated neutrophil/macrophage immune responses and remodelling of the cytoskeleton/ECM, in line with well-established muscle damage > repair processes after mechanically-induced muscle disruption^61,62^. Interestingly, unaccustomed ECC also associated with a suppression of mitochondrial gene networks, implying a role for transient mitochondrial dysfunction in muscle damage > repair, as occurs with ECC-induced mitochondrial swelling in rodents^63^. After habituation to ECC, some of the most prominent transcriptional features that remained relate to upregulated sarcomere assembly genes with putative functions in sarcomerogenesis^64–68^. As such, at least some of the molecular signals putatively driving the ECC phenotype of serial addition of sarcomere units (*versus* CON-related sarcomeres in parallel)^57^ might present immediately and persist as muscle specificity develops, corroborating previous architectural observations^7^. Additionally, ECC was characterised across training by co-ordinated downregulation of a GTPase gene network, with all associated hubs interestingly being TBC1D3 family members, which act as GTPase activating proteins for RAB5^54^. In mammalian cells, both overexpression of the GTP-bound and GDP-bound forms of Rab5 inhibits mTORC1^69^, a key molecular hub regulating muscle anabolism and remodelling^70^. TBC1D3-mediated RAB5 regulation could, therefore, help explain greater ECC-induced anabolic signalling compared to CON^71^.

CON-specific transcriptional responses were substantially lower than ECC, yet clustered to unique functional classes. Since CON training associates with greater metabolic demand^72^ and, consequently, mitochondrial gains *versus* ECC^73^, it is unsurprising that consistent upregulation of mitochondrial genes underscored the untrained and habituated CON response. Further supporting higher CON metabolic strain, an anion transporter-related functional module also increased across training specifically in CON, with a potassium voltage-gated channel (*KCNQ4*) the most highly connected gene in this network. Another striking feature of the CON response was downregulation of ribosome-related networks only in habituated muscle. While perhaps counterintuitive given the importance of ribosomal biogenesis to muscle anabolism and remodelling^74^, these transcriptional profiles possibly reflect enhanced translational efficiency of CON-habituated muscle. Indeed, previous gene profiling of inter-individual variability in exercise adaptations identified an ‘anti-growth’ signature for those with the greatest muscle mass gains that was, in part, characterised by reduced ribosomal RNA gene expression^75^. Our findings also implicate the *RPL7A* component of the 60S subunit as a central, highly connected hub component of such putative translational efficiency, and thus promising mechanistic target for further investigation. Though, whether this response is truly CON-specific, or if ECC performed beyond 2-weeks habituation displays a similar, yet delayed ribosomal suppression remains to be determined.

Congruent with previous work^9,49^, large overlap was found across ECC and CON responses, here including angiogenesis, steroid hormone, transmembrane transporter and DNA methylation/histone regulation pathways. However, whilst small contraction mode-specific profiles were previously observed in young^9,49^, the magnitude of unique ECC/CON profiles are considerably larger in our current work. This discrepancy is likely explained by divergent exercise protocols, since both studies report similar volunteer characteristics, training status, bioinformatic pipelines, gene set identification and biopsy sampling. Specifically, the current use of 30-min graded running is mechanically and metabolically distinct from 70 isolated contractions at 80% maximal effort, as employed previously^9^. A greater overall workload herein might, therefore, explain larger contraction-specific gene responses. Alternatively, our between-group study design introduces extra scope for inter-individual heterogeneity, potentially exaggerating transcriptional differences. Importantly, despite differences in absolute numbers, considerable overlap exists between datasets. For example, 41% of genes commonly regulated by unaccustomed ECC and CON previously^9^ were here too, and 6 of 10 previous ECC/CON modules^49^ map to similarly regulated modules herein. Overlapping hub genes for general contraction responses (LIM-domain and zinc-finger containing genes), or ECC-specific responses (myogenic factors), also provide strong candidates for future examination of general exercise or contraction mode-specific muscle regulation.

Myokines might present as promising therapeutic targets for optimising exercise training adaptations^23,34^. Predictive mRNA-based secretome analysis^22,23^ detected three contraction responsive modules as secretome-enriched pathways in exercised muscle, including one ECC-specific but none CON-specific. Moreover, 30 of the 34 putative myokine hub genes were derived from ECC-specific modules. The muscle secretome might, therefore, exhibit heightened sensitivity to the greater mechanical strain associated with ECC. In this context, the putatively muscle-elevated secretory factor *THBS4* might provide a particularly interesting target for future mechanistic investigation. Indeed, THBS4 has strong roles in regulating muscle structural integrity^56,76^, and its identification as an unaccustomed ECC myokine hub might suggest that THBS4 acts predominantly in an autocrine/paracrine manner to facilitate muscle remodelling upon mechanically-induced disruption. Nevertheless, individuals who experience a more substantial cycle training-induced *THBS4* upregulation in muscle also display the highest improvements in cardiorespiratory fitness (V̇O_2max_)^77^, suggesting that the exercise adaptive role(s) of muscle THBS4 could also extend more globally. Supporting this notion, proteomic analysis of human plasma found increased THBS4 following high intensity endurance exercise^78^. Disentangling the local and systemic function(s) of muscle THBS4 across the entire exercise continuum may thus provide interesting mechanistic insights.

Consistent with higher CON rates of metabolism, the sole CON-specific hub myokine candidate, *VEGFA*, has roles in muscle metabolic homeostasis and insulin sensitivity^79^ as well as regulating angiogenesis^80^. Moreover, *VEGFA* and a striking number of ECC-specific hub myokine candidates have links to ERK/MAPK signalling^81–92^. The ERK/MAPK pathway has been proposed as a potential ECC-focussed mediator of contraction mode-dependent muscle remodelling patterns^7,57^. Given the association across ECC and CON, albeit via distinct secretome components, these results might indicate ERK/MAPK signalling as an important, myokine sensitive/inducing factor in the early stages of training-induced muscle remodelling. A prominent hub myokine candidate across both contraction modes in untrained and habituated muscle was *ANGPTL2*. This proinflammatory gene is identified post-exercise in untrained and trained muscle by unbiased secretome analysis^22,23^, and periodic *ANGPTL2* elevations across exercise training likely support muscle remodelling via myogenic and angiogenic regulation^93,94^. *ANGPTL2* might, therefore, represent a promising myokine biomarker of generalised muscle exercise adaptation. Lastly, all three secretome-enriched modules were under predicted transcriptional control of SP1 and KLF4. While data on myokine regulation by KLF4 is scarce, recent work implicates SP1 as a primary TF responsible for regulating skeletal muscle mRNA levels of established myokines IL-6 and LIF^95^. Extending, SP1 and KLF4 might, therefore, serve as key global regulators of muscle secretome-related contraction responses.

A caveat of our study is the possibility of residual signal(s) in the final post-habituation biopsy from prior ECC/CON bouts, and future work would benefit from the inclusion of an added basal muscle biopsy post-habituation (i.e., prior to the final, post-habituation exercise bout) plus additional control groups (i.e., participants that perform the first and final ECC/CON bouts without 6 training sessions in-between) to fully delineate any potential confounding molecular signals across bouts. Moreover, our analysis of whole muscle tissue homogenate precludes identification of the precise cellular origin of identified signals, which may be overcome in future studies by combining single-cell and spatial transcriptomics. This may be particularly fruitful in the context of our secretome analyses, where delineating the precise cell-type location of identified myokine targets could provide additional clues on their secretion status and associated intramuscular and/or multi-tissue crosstalk mechanism(s) in a contraction mode-(in)dependent manner.

In summary, we combined global transcriptomic profiling with network analysis to explore molecular signatures and predict molecular candidates of muscle adaptation during early habituation to ECC *versus* CON training. Exercise habituation attenuates transcriptomic responses, the molecular functional profiles of which do not substantially diverge from untrained responses to either contraction mode. However, new insights into key contraction mode-specific molecular differences are demonstrated that might plausibly account for known phenotypic differences during habituation to ECC *versus* CON. Network-driven data reduction approaches also produce a tractable list of promising candidate molecules, including novel candidate myokines, that might help explain phenotypic differences during exercise habituation to ECC *versus* CON. As such, this study establishes a strong platform from which to expedite mechanistic understanding and optimisation of muscle/whole-body adaptations to exercise training.

## Supplementary Information

Supplementary Information 1.

Supplementary Information 2.

Supplementary Information 3.

Supplementary Information 4.
