# Supplementary material for: Transcriptomic adaptation during skeletal muscle habituation to eccentric or concentric exercise training
Source: Sci Rep. 2021 Dec 14;11:23930. doi: 10.1038/s41598-021-03393-7 (PMC8671437; doi:10.1038/s41598-021-03393-7)
Supplement: Supplementary file 1 — Supplementary Information 1. [file 41598_2021_3393_MOESM1_ESM.docx]

Supplementary Information

Supplementary File 1 contains gene-level transcriptomic analyses. Supplementary File 2 contains network-level transcriptomic analyses. Supplementary File 3 contains network-driven secretome analysis data.
